# Supplementary material for: Quantification of human mature frataxin protein expression in nonhuman primate hearts after gene therapy
Source: Commun Biol. 2023 Oct 27;6:1093. doi: 10.1038/s42003-023-05472-z (PMC10611776; doi:10.1038/s42003-023-05472-z)
Supplement: Supplementary file 2 — Description of Additional Supplementary Files [file 42003_2023_5472_MOESM2_ESM.pdf]

Description of Additional Supplementary Files

File name: Supplementary Data 1
Description: Numerical source data for the graphs in Figures 2B and 2C.

File name: Supplementary Data 2
Description: Numerical source data for the graphs in Figure 5E.

File name: Supplementary Data 3
Description: Numerical source data for the graphs in Figure 7.

File name: Supplementary Data 4
Description: Numerical source data behind Table 2A.

File name: Supplementary Data 5
Description: Numerical source data behind Table 2B.
